# Three-dimensional geometric morphometric studies of modern human occipital variation

**DOI:** 10.1371/journal.pone.0245445

**Published:** 2021-01-14

**Authors:** Yameng Zhang, Lynne A. Schepartz

**Affiliations:** 1 Joint International Research Laboratory of Environmental and Social Archaeology, Shandong University, Qingdao, China; 2 Institute of Cultural Heritage, Shandong University, Qingdao, China; 3 Faculty of Health Sciences, Human Variation and Identification Research Unit (HVIRU), School of Anatomical Sciences, University of the Witwatersrand, Johannesburg, South Africa; 4 University of Pennsylvania Museum of Archaeology and Anthropology, Philadelphia, Pennsylvania, United States of America; The Cyprus Institute, CYPRUS

## Abstract

**Objectives:**

To investigate three-dimensional morphological variation of the occipital bone between sexes and among populations, to determine how ancestry, sex and size account for occipital shape variation and to describe the exact forms by which the differences are expressed.

**Methods:**

CT data for 214 modern crania of Asian, African and European ancestry were compared using 3D geometric morphometrics and multivariate statistics, including principal component analysis, Hotelling’s T^2^ test, multivariate regression, ANOVA, and MANCOVA.

**Results:**

Sex differences in average occipital morphology are only observed in Europeans, with males exhibiting a pronounced inion. Significant ancestral differences are observed among all samples and are shared by males and females. Asian and African crania have smaller biasterionic breadths and flatter clivus angles compared to Europeans. Asian and European crania are similar in their nuchal and occipital plane proportions, nuchal and occipital angles, and lower inion positions compared to Africans. Centroid size significantly differs between sexes and among populations. The overall allometry, while significant, explains little of the shape variation. Larger occipital bones were associated with a more curved occipital plane, a pronounced inion, a narrower biasterionic breadth, a more flexed clivus, and a lower and relatively smaller foramen magnum.

**Conclusions:**

Although significant shape differences were observed among populations, it is not recommended to use occipital morphology in sex or population estimation as both factors explained little of the observed variance. Other factors, relating to function and the environment, are suggested to be greater contributors to occipital variation. For the same reason, it is also not recommended to use the occiput in phylogenetic studies.

## 1. Introduction

The occipital bone is composed of squamosal, basilar and two lateral portions that develop via two forms of ossification. Intramembranous ossification of the squamosal portion or occipital plane is a direct formation process from mesenchymal tissue into bone and involves at least two pairs of ossification centers. Endochondral ossification of the other portions is an indirect bone formation process involving the mineralization of cartilage [1–3].

**Fig 1:**
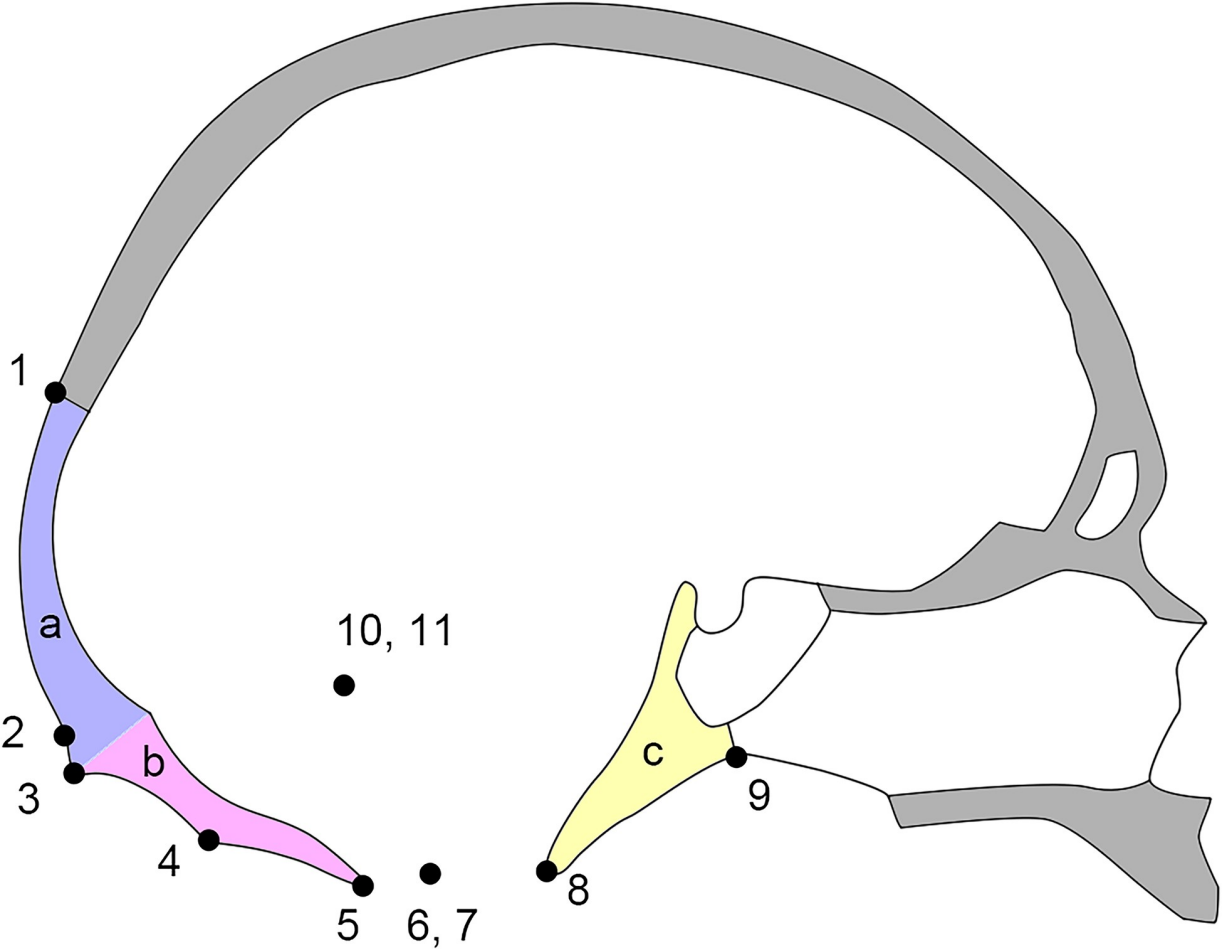
Diagram of occipital landmarks and occipital components in sagittal profile. 1. Lambda, 2. Suprainion, 3. Inion, 4. Intersection of the inferior nuchal line and external occipital crest, 5. Opisthion, 6, 7. Left and right occipital postcondylar point, 8. Basion, 9. Hormion, 10, 11. Left and right asterion, a. Occipital plane, b. Nuchal plane, c. Occipital clivus.

The adult occipital shape is generally described in terms of two planes and their junction: the superiorly positioned occipital plane and the inferiorly positioned nuchal plane (Fig 1). The nuchal musculature attaching on the surface of the nuchal plane includes the sternocleidomastoid, trapezius, semispinalis capitis, rectus capitis posterior major, rectus capitis posterior minor and obliquus capitis superior muscles. Histologically, the deep nuchal muscles possess a high density of proprioceptors that likely perform mechanosensory rather than mechanical functions related to movement [4–6].

The occiput has been widely used in sex estimation in forensic anthropology. Some studies demonstrated the efficacy of the size of foramen magnum or occipital condyles for sexing [7–10], while others questioned the universal value of metrical analyses for sex estimation [11–14]. In fact, many studies lacked blinded testing, cross-validation procedures or statistically reliable results [15,16]. Consequently, sex estimation still relies greatly upon the assessment of nonmetric features such as the nuchal line, nuchal crest, occipital torus and external occipital protuberance (EOP) [17–19]. The classic method in Buikstra and Ubelaker [20] uses a combination of five discrete traits including the robustness of the nuchal crest, which was demonstrated to be useful in later studies [21–23]. Nevertheless, the use of traditional non-metric methods raises several concerns, such as the difficulty in quantifying nonmetric traits, non-comparable results due to different scoring methodologies across studies and high degrees of interobserver error [24,25]. Also, potentially useful information regarding the subtle expression of discrete traits and shape differences is inevitably lost during the process of non-metric scoring.

Geometric morphometrics has been applied in many fields to explore shape variation beyond what is possible with traditional metric analyses. Technical advances expanded the application of 3D geometric morphometrics due to the convenient and rapid acquisition of 3D landmark data [26–29]. When compared to traditional morphometrics, geometric morphometric methods are greatly advantageous in that they quantify shapes with landmark coordinates, and shape changes can be visualized easily with the help of deformation grids or relative displacements of corresponding landmarks [30]. In an early study, Ahlström [31] employed 42 3D landmarks covering most of the cranium to reveal significant sex differences, whereby only males exhibited posterosuperior inion deformations. The main sexually dimorphic cranial deformations that were identified did not involve lateral dimensions, but were limited to anteroposterior and superoinferior dimensions that were attributed to constraints imposed by mastication and nuchal muscle functions. Wood and Lynch [32] observed significant sex differences in African, but not European crania, using nine midsagittal landmarks. Subsequently, Rosas and Bastir [33] found that European males exhibited pharyngeal expansion and a downward pointing occipital clivus. More recently, Bigoni, Velemínská and Brůzek [34] found that European males had a more posteriorly projecting occipital plane and also observed significant sex differences involving separate areas of the cranium; however, they failed to confirm this result using the whole cranium and recommended that local area analysis should be used instead. Notably, a series of studies focusing on South African crania found significant sex differences for the frontal bone profile, supramastoid crest, prognathism and posterior airway space [35–38]. Some of the shape changes were suggested to relate to muscle development.

Allometry, the study of how biological shape changes with size, is also considered in the present study. There are three types of allometry: evolutionary, ontogenetic and static allometry [39,40]. Current studies of occipital allometry mainly focus on the rotation of the cranial base. From a developmental perspective, the cranial base flattens out by some 15–20° during ontogeny [41]. Some suggest this as an adaption to brain expansion [42] while others doubt this explanation [43,44]. From an evolutionary perspective, humans have a highly flexed cranial base compared to non-human primates. Several hypotheses have been suggested to explain this and chief among these are the spatial packing hypothesis [45,46] and the bipedalism or postural hypothesis [47–49]. The spatial packing hypothesis suggests that the cranial base anterior to the sella turcica is deflected downward and the post-sella cranial base is rotated anteriorly to accommodate the expanding brain [50]. Further evidence from experimental mouse studies, human ontogenetic research and artificial cranial deformation studies lends support to the importance of the spatial packing hypothesis as a driver of basicranial flexion characteristics [50–54]. The postural hypothesis posits that the occipital condyles and posterior part of the cranial base are rotated anteriorly to maintain the face in a forward-looking position during orthogonal posture [50,55–57].

Considering the insufficiency of studies on sex and ancestry differences based on 3D occipital morphology and ambiguity regarding how allometry and brain expansion relate to occipital diversity, this study aims to investigate 3D occipital shape variation among and within samples from major world regions (Africa, Asia and Europe) to determine how ancestry, sex and size account for occipital shape variation, and to characterize how the differences are expressed.

## 2. Materials and methods

### 2.1 Sample characteristics

Using micro computed tomography (micro-CT), 214 dry crania were scanned. All specimens were adults of known sex with a good state of preservation. Seventy were Asian crania obtained from the Institute of Vertebrate Paleontology and Paleoanthropology (IVPP) in Beijing. Both recent northern and southern Chinese specimens were used to represent Asian regional diversity. The southern Chinese are from a public graveyard founded in the 1820s and collected by Woo Ting-Liang in the 1940s [58,59]. The northern Chinese crania are from several different historic proveniences. A total of 144 African and European crania were selected from the Raymond A. Dart Collection of Modern Human Skeletons, housed in the School of Anatomical Sciences, University of the Witwatersrand (Wits) (Table 1). The Africans and Europeans from this collection are primarily cadavers collected from local Johannesburg hospitals. Basic demographic information is documented in the Dart Collection catalog [60]. The Europeans are representative of the groups (primarily Dutch and English) who migrated to South Africa over the past several hundred years. The African skulls represent the majority population of South Africa and include several Bantu language-speaking ethnic groups (including Soto, Zulu, Xosa, Pedi, and Tswa). They are treated here as a single group as morphological and genetic studies determined that the differences among these groups are very small because of recent admixture [61–64]. The Dart Collection was used in many previous geometric morphometric studies addressing different questions [37,38,65,66].

**Table 1:** Sample composition.

	Male	Female	Total	Source
Africa	28	34	62	Wits
Asia	38	32	70	IVPP
Europe	37	45	82	Wits
Total	93	121	214	

The Asian crania were scanned at the IVPP using a scanning resolution of 160 μm followed by transformation to tomographic slices, each with an original image size of 2048×2048 pixels. The African and European cranial images were processed by the micro-CT scanner at the Microfocus X-ray Computed Tomography (CT) Facility at the University of Witwatersrand (Wits) using a default scanning resolution of 130 μm, with higher resolution levels used as needed according to specimen size. Images of transformed tomographic slices in TIFF format were generated with an original image size of 2048×2048 pixels.

Permissions to use cranial specimens were granted by the Institute of Paleontology and Paleoanthropology (IVPP) and the University of the Witwatersrand. Ethical approval for this study was obtained from the University of the Witwatersrand School of Anatomical Sciences (ethical clearance W-CJ-140604-1) under the South African Human Tissue Act No. 65 of 1983 and the National Health Act No. 61 of 2003 that applies for use of the Raymond A. Dart Collection of Modern Human Skeletons in scientific research.

### 2.2 Definition of three-dimensional landmarks

For the geometric morphometric analysis, 11 3D landmarks were chosen to capture the shape of the occipital bone components (Fig 1). Both Bookstein type I and type II landmarks were used in this study. Lambda, inion, the intersection of the inferior nuchal line and the external occipital crest, hormion, and asterion are Bookstein type I landmarks as they are intersections between bony sutures or crests. Suprainion, opisthion, occipital postcondylar point, and basion are Bookstein type II landmarks that are local minima and maxima of curves [67,68].

### 2.3 Geometric morphometrics

In geometric morphometrics, generalized Procrustes analysis (GPA) is performed to separate size and shape components. Shape is defined as “the geometric information that remains when location, scale and rotational effects are filtered out from an object” [69]. Shape variables, also referred to as the Procrustes coordinates, are separated after GPA. The average shape is calculated as a representation of the group mean that can be used for subsequent intergroup comparisons. To test how shape variables are influenced by sex, population, centroid size and their interaction terms, a full Procrustes MANCOVA model is built using Procrustes coordinates as dependent variables, while sex, population, the interaction term between sex and population, the interaction term between sex and centroid size, the interaction term between population and centroid size are used as independent variables, and centroid size as a covariate. The interaction term between sex and centroid size (p = 0.253), and the interaction term between population and centroid size (p = 0.285) were nonsignificant and removed in the formal analysis. Multiple comparisons of the average occipital shape between sexes and among populations were then tested by Hotelling’s T-squared statistics with a permutation test (n = 1000) and the p value were adjusted through the Benjamini-Hochberg p-value adjustment (the “BH” method) [70,71].

Principal component analysis is performed to summarize multivariate data by building linear combinations of the original shape variables, which are the Procrustes coordinates here. Then the specimens are projected into the shape space created by PC scores. Together with the group label, the distribution of the different groups in the shape space can be visualized directly. At the same time, occipital shape changes along the different PCs can be determined using wireframe landmark displacements.

Centroid size (CS) for a set of landmarks is the square root of the sums of squared distances between landmarks and their centroid and is used to represent the overall size of the landmark configuration. For the allometric study, this analysis uses the common allometric component score (CAC score) or the regression score, which separates shape with size to test the significance of any detected allometry [72]. Also, by decomposing the Procrustes variation, the extent that size explains shape change can be determined [72–74]. Multivariate regression is first performed to test the existence of allometry within each population in males and females. MANCOVA is then performed using Procrustes coordinates as a dependent variable, using the centroid size as the covariate, and using sex, population, the interaction term between sex and centroid size, and the interaction term between population and centroid size as independent variables with a permutation test (n = 1000) to assess the significance. The analysis was performed in R version 3.6 using the *geomorph*, *shapes*, *tidyverse*, and *ggplot2* packages [75–78].

### 2.4 Intraobserver error

Data were collected by one researcher (YZ) to avoid interobserver error. To assess the intraobserver error, YZ collected 3D landmarks on 12 crania (two crania for each sex and population) three times repeatedly on three different days. The data were then submitted to GPA, Principal Component Analysis and Procrustes MANCOVA. In the scatter plot of the first four PCs, all the repeated data were closely related to each other. In the Procrustes MANCOVA, the group factor (p = 0.96) that comprised the three repeated landmark sets had no significant influence on the shape variables. Therefore, the intraobserver error was considered to be much smaller compared to the sample variability.

## 3. Results

### 3.1 Average shape differences between sexes and among populations

According to the Procrustes MANCOVA (Table 2), significant differences were found both between sexes (p = 0.010) and among populations (p = 0.001). The interaction term between sex and population is approaching significance (p = 0.074), which suggests that sex differences might differ in different populations. According to the explained sums of squares, sex and population accounted for 1.30% and 9.60% of the total variation respectively (calculated by dividing the explained sum of squares by the total sum of squares, namely the R square). Obviously, compared to sex, ancestry plays a more important role in occipital shape morphology. However, it is noteworthy that the unexplained residuals were still the largest portion, indicating great within group variation [79]. This means that factors not included in this analysis contribute more to occipital morphology than sex and ancestry.

**Table 2:** Procrustes MANCOVA with sex, population and centroid size.

	Df	SS	MS	Rsq	F	Z	Pr(>SS)
Sex	1	0.019	0.019	0.013	3.125	2.680	0.010*
Population	2	0.140	0.070	0.096	11.633	7.154	0.001**
Centroid size	1	0.031	0.031	0.021	5.103	3.670	0.001**
Sex:Population	2	0.019	0.009	0.013	1.555	1.440	0.074
Residuals	207	1.247	0.006	0.857			
Total	213	1.456					

Df: Degree of freedom, SS: Sums of squares, MS: Mean of sums of squares, Rsq: R square.

*p<0.05,

**p<0.01.

**Fig 2:**
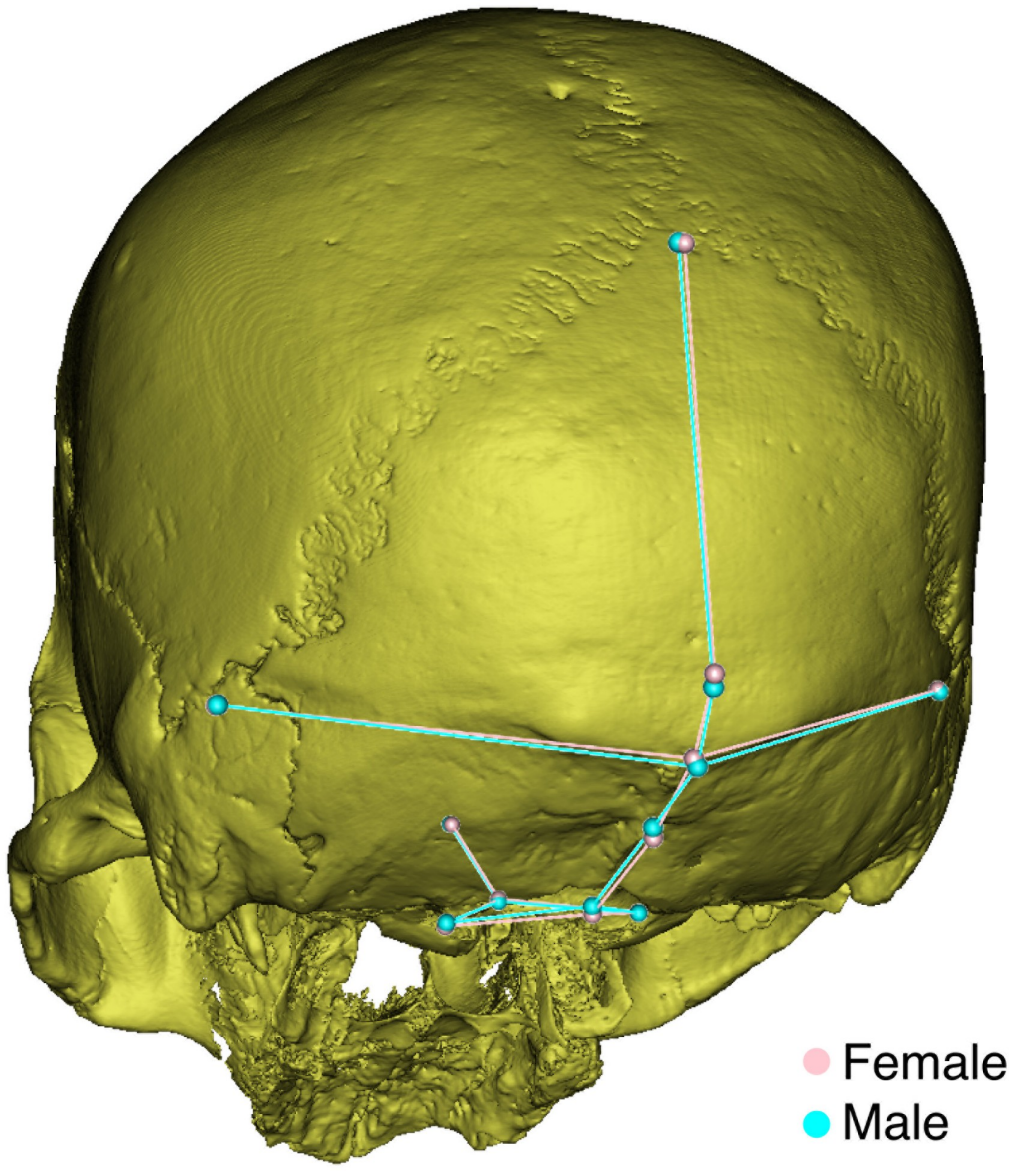
European male and female average occipital shape superimposition.

To further clarify how occipital shape was affected by sex and population, multiple comparisons of average shape were made within males and females and within each sample. Sex differences were not statistically significant in all the populations. They were only found in Europeans (p = 0.004), in contrast to Asians (p = 0.302) and Africans (p = 0.122). The European female and male average occipital shapes were superimposed via wireframes and displaced landmarks to show how the shape differs with sex (Fig 2). European males show a lower inion and suprainion position and the external occipital protuberance is more pronounced than that of females. Males also have a more concave nuchal plane and thus a sharper nuchal-occipital angle.

Significant population differences were observed within male, female and pooled samples separately (Table 3). Population differences were significant in all of the groups, indicating that the ancestral differences were consistent and were independent of sex.

**Table 3:** Pairwise population comparisons within sexes.

	Male		Female		Pooled	
	H	Adjusted p value	H	Adjusted p value	H	Adjusted p value
Asia-Africa	4.760	0.024*	5.380	0.022*	7.030	0.002**
Asia-Europe	4.940	0.024*	3.870	0.021*	8.310	0.002**
Africa-Europe	3.840	0.038*	5.560	0.006**	10.280	0.002**

H represents the Hotelling statistic; p values are adjusted by the “BH” method.

*p<0.05,

**p<0.01.

**Fig 3:**
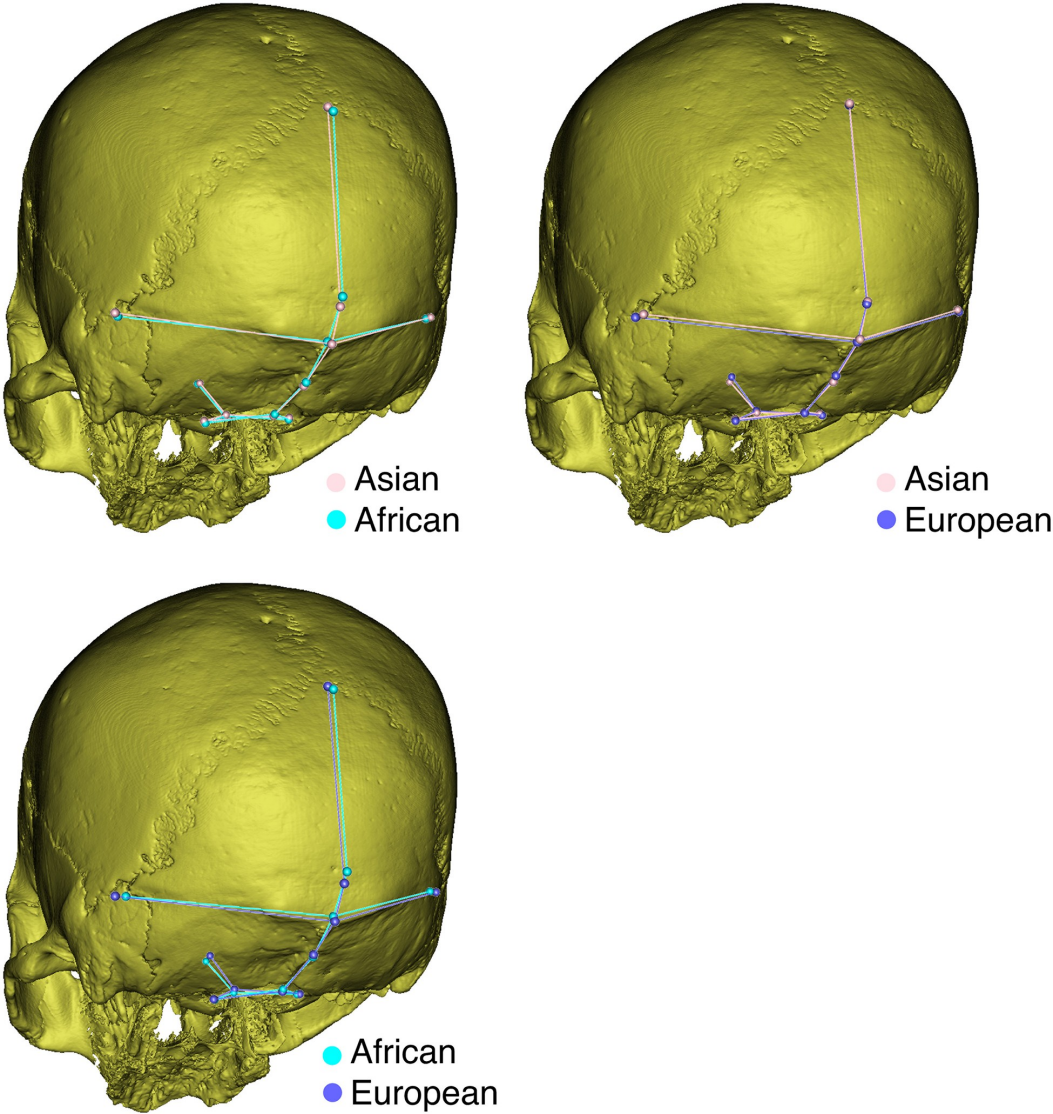
Pairwise population comparisons of average occipital shape.

The average shapes of the three populations are superimposed to show their differences in Fig 5. Asian and African crania resemble each other in the biasterionic breadth that is smaller than that observed for the European crania. They also have a similar clivus angle, which is flatter than what is found in European crania. Asian and European crania are similar in the nuchal and occipital plane proportions, nuchal and occipital angles, and the position of inion, which is lower in the African crania. Compared to the variation among populations (Fig 3), shape variation between populations was much smaller.

### 3.2 Principal component analysis

**Fig 4:**
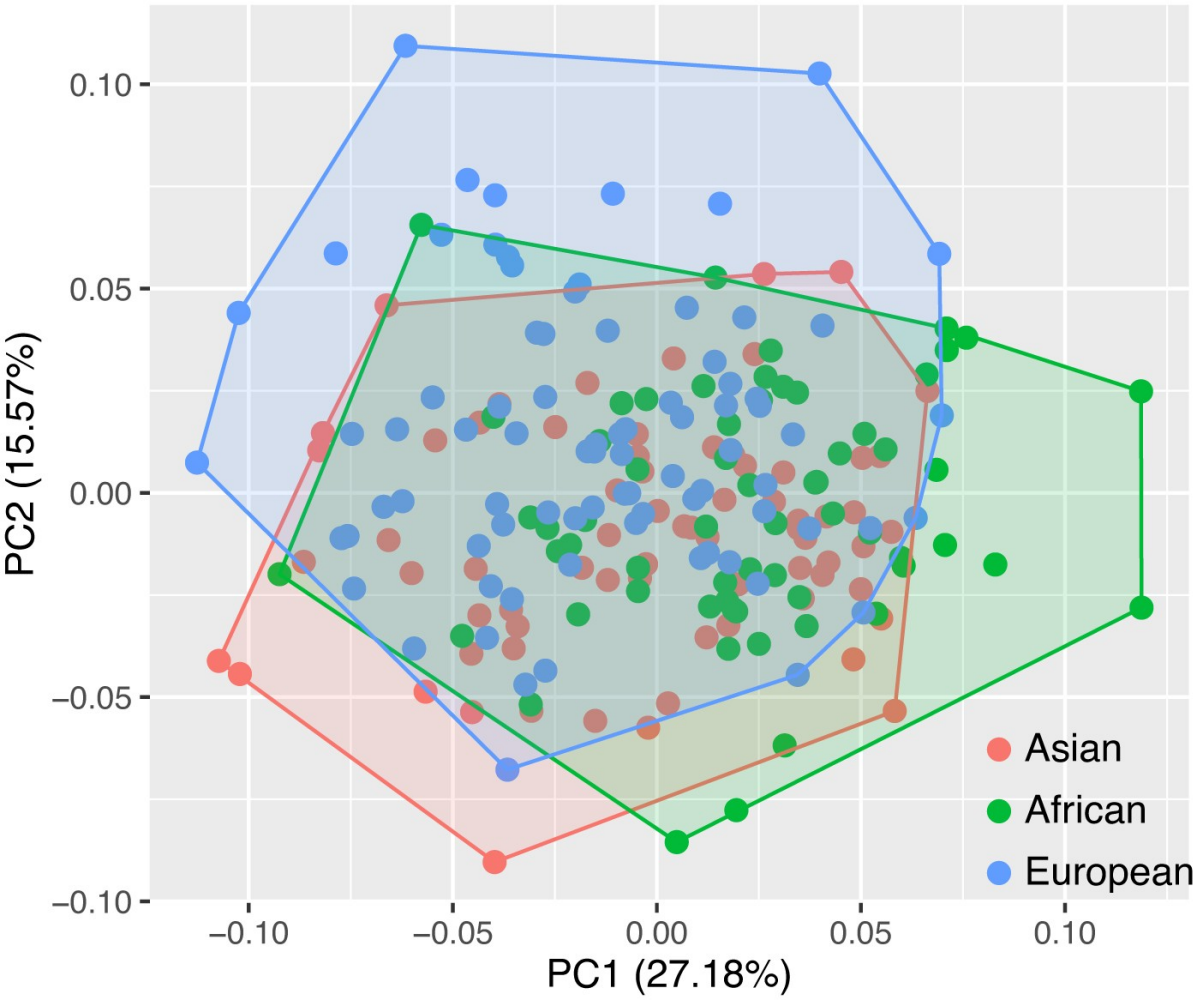
Scatterplot of the first two principal components.

Sex differences were only observed in the European sample, and sex explained less of the variance than ancestry in the MANCOVA. Therefore, principal component analysis was carried out only using the population as a label (Fig 4). A PC is regarded as meaningful if the ratio of this PC and its successor is above a threshold based on a log-likelihood ratio [80,81]. According to the PCA, the meaningful PCs were PC1 and PC2, which explain 42.57% of the variation. There is no clear separation among ancestries on both PC1 and PC2. The shape changes along PC1 and PC2 are shown by landmark superimpositions (Fig 3).

**Fig 5:**
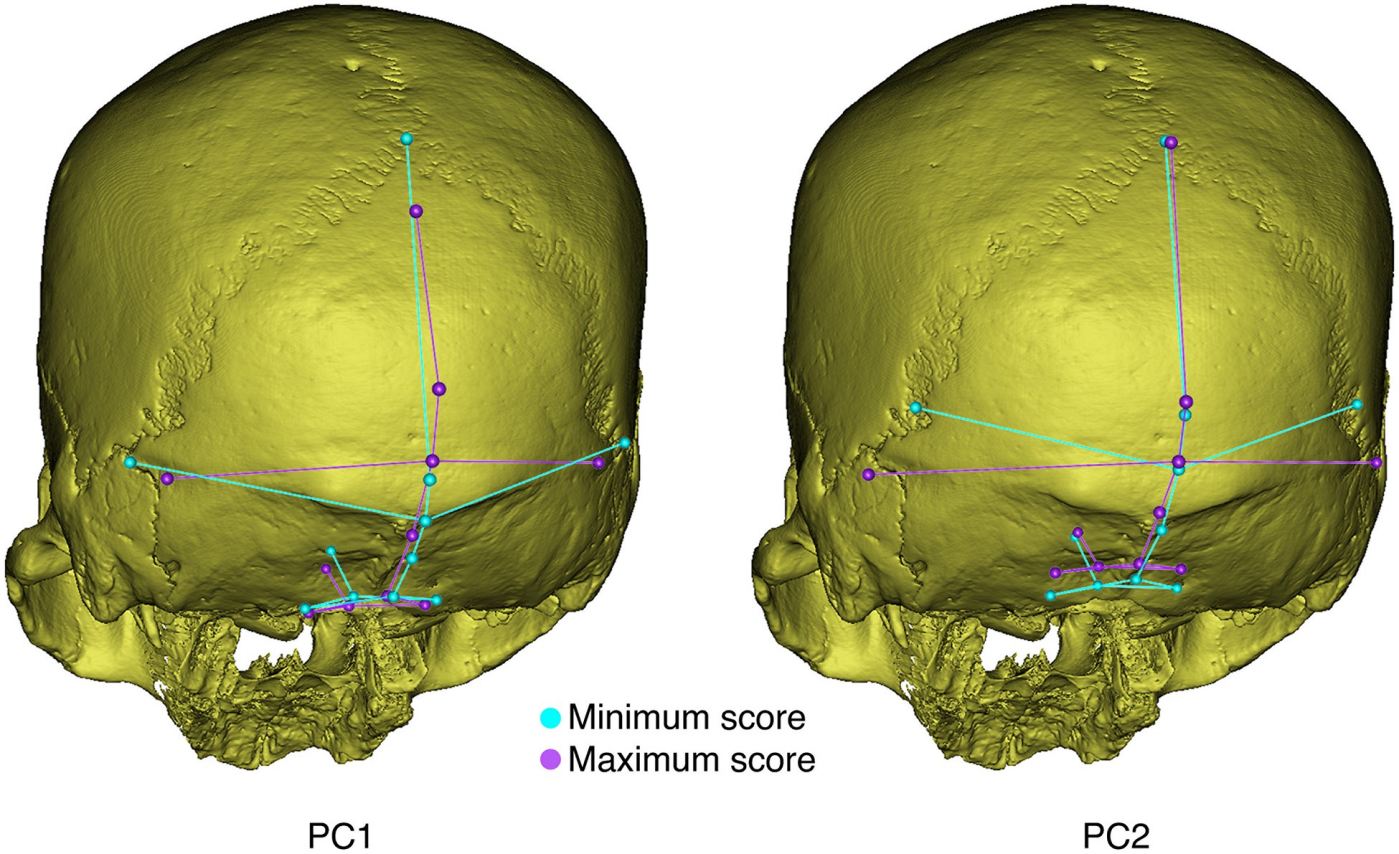
Shape change along PC1 and PC2.

For PC1, occipital shape differs mainly in the relative proportions of the occipital and nuchal planes (Fig 5). In this analysis, along the negative direction of PC1, the occipital plane is larger than the nuchal plane, the inion is in a very low position, the clivus is higher, and the asterion is wider. Regarding PC2, the occipital shape changes markedly at asterion, which involves the anteroposterior depth, medial-lateral breadth and vertical height (Fig 3). Along the positive direction of PC2, individuals have more anteriorly positioned, wider and lower asterions.

### 3.3 Centroid size and allometry

**Fig 6:**
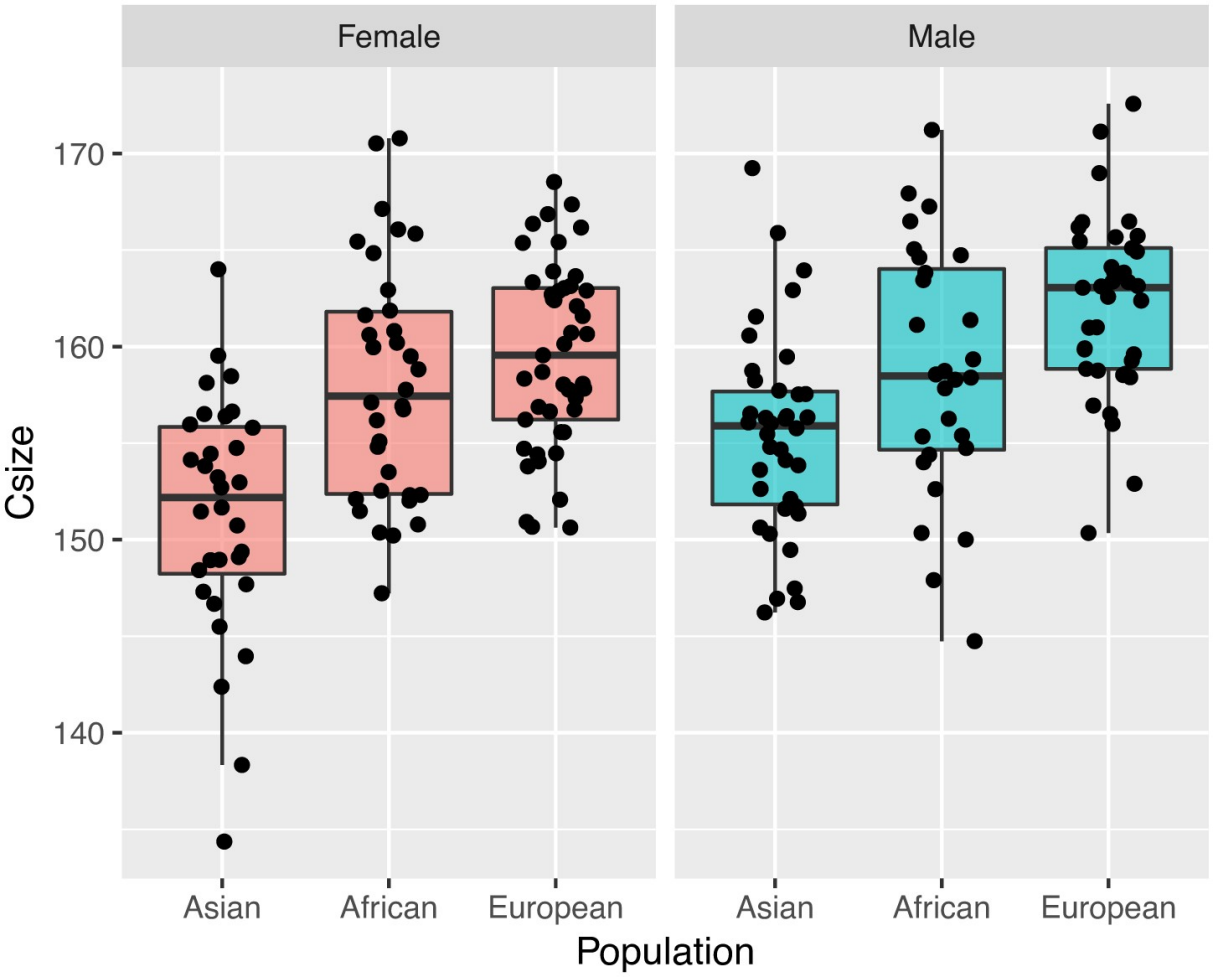
Plot of centroid size in different sexes and ancestries.

Using centroid size as the dependent variable, a two-way ANOVA revealed significant sex differences (p = 0.024) and population differences (p = 0.001) without interaction (p = 0.160). Males (158.73) have a larger centroid size than females (156.75) in each sample. Overall, ancestral differences are also consistent with differences observed within male and female groups, with centroid size ranked from largest to smallest from Europeans (160.72) to Africans (158.40) to Asians (153.56). This pattern is exhibited in the box-whisker plot (Fig 6).

Allometry was first tested within each population in males and females with multivariate regression using Procrustes coordinates and centroid size. Males and females were different in their allometric patterns in all of the samples: Asian males (p = 0.148, R^2^ = 0.039) and females (p = 0.015, R^2^ = 0.099), African males (p = 0.017, R^2^ = 0.087) and females (p = 0.073, R^2^ = 0.051) and European males (p = 0.017, R^2^ = 0.060) and females (p = 0.416, R^2^ = 0.022). Africans and Europeans are similar as they have significant allometry in males instead of females. Based on the R squares, the relationship is very weak through all the groups.

**Fig 7:**
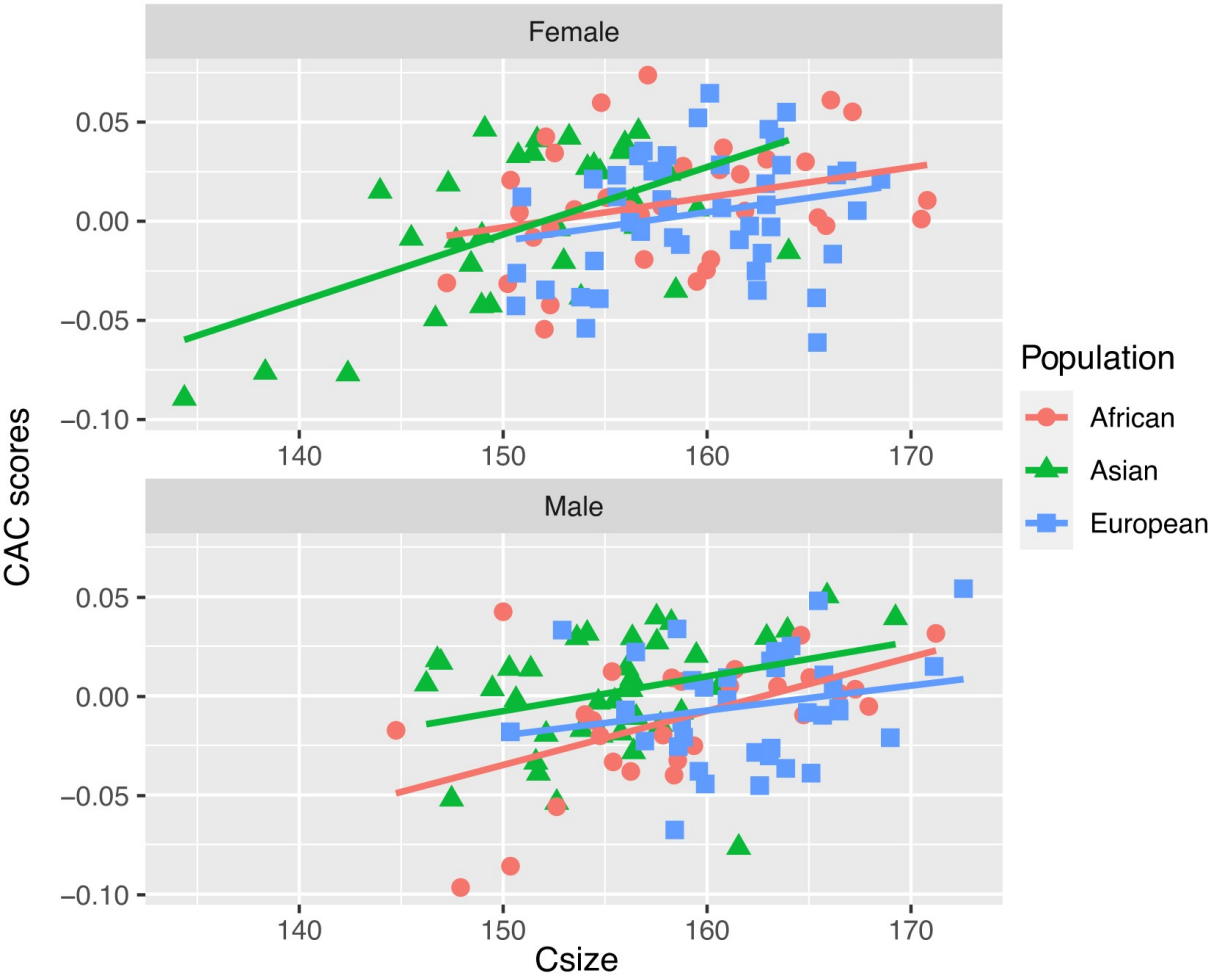
Multivariate regression with Procrustes coordinates and centroid size.

The Procrustes MANCOVA (Table 2) performed previously revealed significant overall allometry (p = 0.001, R^2^ = 0.021), with the centroid size contributing 2.1% to the shape variation. The allometric pattern determined by the interaction term (slope) does not differ significantly between sexes (p = 0.253) or among populations (p = 0.285). A scatterplot with CAC scores and centroid size among populations with males and females separately was drawn to show the sexual and ancestral allometric patterns (Fig 7).

**Fig 8:**
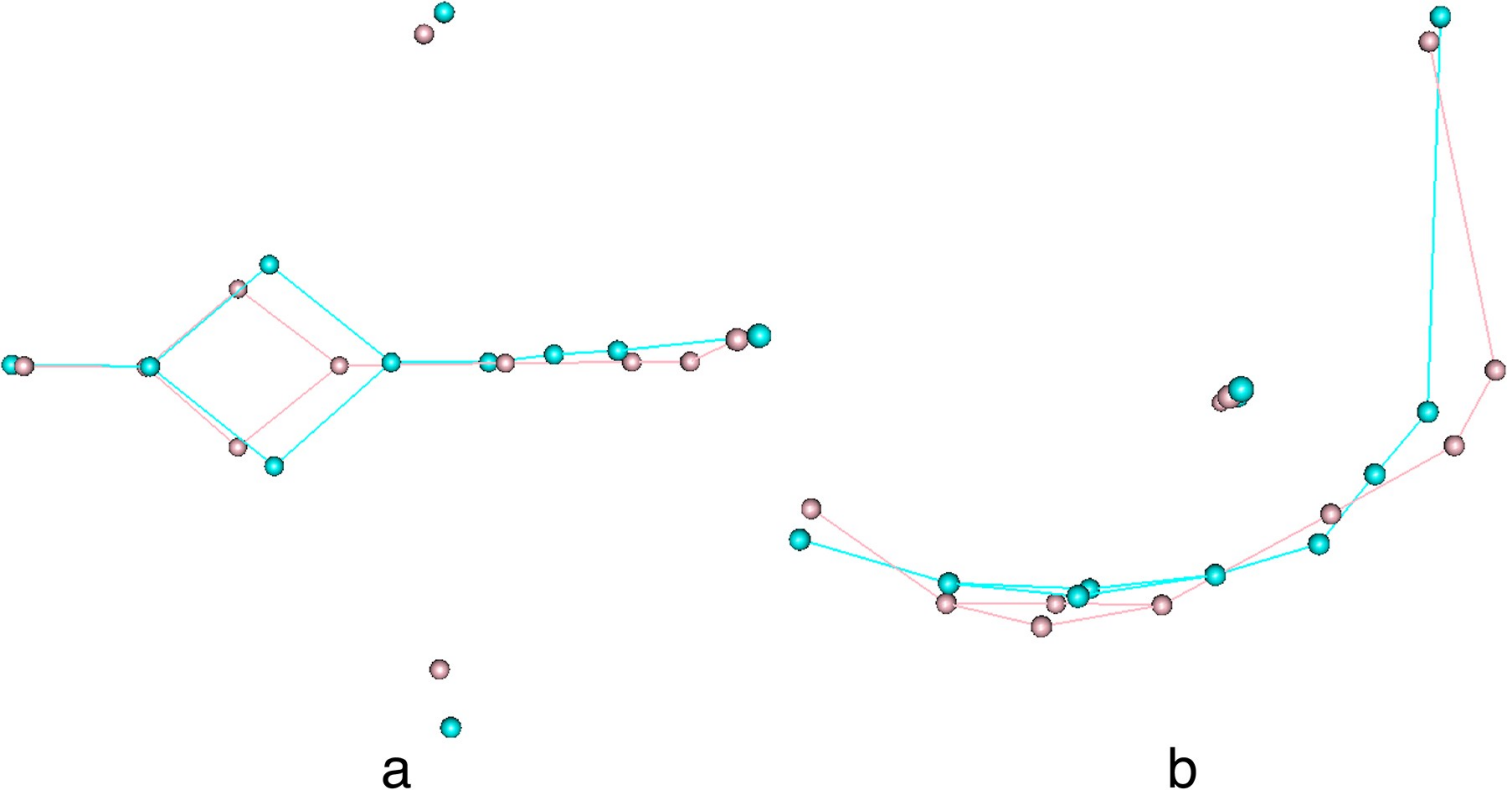
Occipital shape from minimum (cyan) to maximum (pink) size. a, superior view, b, lateral view.

Because the allometric pattern is overall significant and does not differ significantly between sexes and among populations, shape changes were plotted using all the samples. Occipital shape superimposition from minimum to maximum centroid size is drawn (Fig 8). A larger occipital bone is accompanied by marked curvature with a more rotated occipital clivus, a higher and pronounced inion, and narrower biasterionic breadths. The allometric trend characterizes curvature in both the sagittal and axial planes, as well as the relative proportions of the nuchal and occipital planes. Notably, the relative size of the foramen magnum in small occipital bones is enlarged and its position is higher than in the large occipital bones.

## 4. Discussion

### 4.1 Three-dimensional morphological variation of the occipital bone

Principal component analysis revealed that major occipital shape variation involves the area surrounding inion, and leads to changes in the relative proportions of the nuchal and occipital planes. Variation also occurs in the axial and sagittal directions, where shape change is more strongly associated with the asterionic position relative to the inion. By contrast, compared to the aforementioned variation, the shape of the foramen magnum and clivus are relatively stable across the samples. This is in concordance with a previous study by Olivier [82] that found the basilar part is biometrically independent from the occipital squama. Olivier also suggested that the occipital squama is a stable anatomical structure despite its dual histological basis. However, this is not supported by our study. Bone remodeling of the inion region is very active until adolescence and is heavily influenced by environmental stresses and behavior [83–85]. Thus, the inion region varies more than other region of the occipital morphologically, as documented in this study.

### 4.2 Sex and population differences

Significant occipital sex difference was observed in the pooled sample and the Europeans, but it was absent in the other samples. This is in agreement with previous studies that found the occipital was not useful for sex estimation [34,61,86,87]. Although Gulekon and Turgut [88] demonstrated significant sex differences in central Anatolian samples using the chi-squared test, they realized that the small developmental variation observed in the EOP between males and females overlaps to a great degree, making EOP variation unsuitable for use in sex estimation.

Furthermore, certain behavioral variations can affect occipital morphology in ways that may obscure sex and population differences. For example, recent studies have shown that the forward head protraction (FHP) induced by poor postures (stemming from heavy use of computers and handheld mobile devices) can cause repetitive and sustained mechanical loads on the neck muscles, thus contributing to a high prevalence of enlarged external occipital protuberance (EEOP) in young adults [85,89].

Compared to the shape of the EOP, the roughness of an area or crest caused by muscle attachment gives more information on relative strain [90]. This is in concordance with the fact that most of the deep nuchal muscles are characterized by a high density of muscle spindles for precise movement of the head and neck, as seen in the capitis rectus posterior minor, capitis rectus posterior major and obliquus capitis superior muscles; these tissues are not designed for generating higher forces that can affect the shape of the occipital bone [4–6].

Current study revealed a flatter occipital curvature of Europeans and Asians compared to Africans. Regarding to the cranial base flexion, Rosas and Bastir [91] found that the anterior cranial base in Africans is rotated in a forward-downward position while in Asians and Europeans it is rotated in the opposite way. However, the posterior cranial base (clivus) shows a different pattern, which is flatter and smaller in Europeans while flexed and larger in Asians and Africans [91,92]. Contrary to their findings, we found that Europeans have the greatest clivus flexion, while Asians and Africans are flatter. However, the posterior cranial base occupied only a small proportion of the shape variation compared to the face, the maxilla and the anterior cranial base in their study [91,92]. Therefore, the discrepancy with their study may be subtle and due to population variation within European samples as the similarity between Asian and African populations was the same.

### 4.3 Allometric pattern

A larger occipital bone was associated with a more curved squamosal shape and a rotated clivus (resulting in basicranial flexion) with biasterionic breadth reduction. The narrow shape of a large occipital bone could be explained by spatial compensation for a longer occipital bone [53], and the basicranial flexion observed in this study fits the spatial packing hypothesis. However, Rosas and Bastir [33] found that larger crania are accompanied by a flatter clivus, which seems to be in contradiction with previous studies. They also observed a marked shift in the proportions of the neurocranium and viscerocranium along with size. As they were dealing with more facial and mandible landmarks, the flatter clivus is actually the result of a larger viscerocranium, which corresponds to a small neurocranium. A later study also demonstrated that cranial base flexion not only relates to the expansion of the cranium but also to facial size [93]. These findings illustrate the importance of choosing a proper size agent and identifying the module in the study of allometry.

How foramen magnum size changes relative to the occipital bone has not been well studied previously. Gruber and colleagues [12] posited that the size of the foramen magnum would not differ by age, sex or secular trend. Other than that study, the most closely related investigations are those on the allometry of the spinal cord. Although the commonly accepted brain-body size scaling exponent in mammals is 0.75 [94], this is not the case for the spinal cord. Fox and Wilczynski [95] studied the allometric pattern of major central nervous system (CNS) divisions and calculated the average cross-sectional area of the cervical spinal cord (SCA). The exponent with body weight is less than 0.66 in most mammals. Furthermore, in the study of the scaling of the gross dimension of the spinal cord to body weight in primates, MacLarnon [96] obtained an exponent of 0.69, which is significantly lower than the scaling exponent of 0.75 for brain weight to body weight. In this study, the size of the occiput and foramen magnum can represent the brain size and the spinal cord to a certain extent [96]. The greater allometric trend of the foramen magnum observed here is consistent with the previous studies on the spinal cord. Although the brain-body size relationship in mammals has been explained by a variety of hypotheses (i.e., social [97], ecological [98], energetic [99], life history [100], and behavioral perspectives [101]), the reason why the spinal cord has an even lower scaling exponent to body weight (a more negative allometric pattern) is still not clear. This result needs to be further investigated and verified in a larger sample.

### 4.4 Limitations

In this study, we did not apply semilandmarks since the overall occipital configuration was our major interest. Also, compared to landmarks, semilandmarks often lack the property of homology [68]. However, the usefulness of semilandmarks in capturing the shape of non-metrical features should not be dismissed, i.e., in the analysis of the shape of nuchal lines and the external occipital protuberance. Also, applying semilandmarks will help achieve a balanced overall landmark configuration of the occiput. Adding semilandmarks in a future study has great potential for investigating the shape of occiput.

This study used crania from three major world regions to investigate occipital shape variation. The selected samples do not capture all the variation for those regions or modern humans overall. For those reasons, the results may be limited in their generalizability. Moreover, both sex and ancestry explained little of the total variance, implying more important factors are acting on occipital shape, such as the process of ontogeny, functional adaptations, and environmental conditions. Using specimens of different developmental stages, populations with different subsistence strategies, or adding climate factors to the analysis will shed light on this topic. Despite this, the results show distinctly different patterns of sex difference and morphological variation across the regions that support the importance of geometric morphometric analyses for identifying patterns and generating future research questions.

## Supporting information

S1 File (TPS)

S2 File (TPS)

S3 File (TPS)
